# One-Two Punch: Phage-Antibiotic Synergy Observed against *Staphylococcus aureus* by Combining Pleurotin and Phage K

**DOI:** 10.1021/acsomega.4c09831

**Published:** 2025-03-18

**Authors:** Michaël Dagne Tadesse, Nala Ali, Martha White, Lijiang Song, Fabrizio Alberti, Antonia P. Sagona

**Affiliations:** † School of Life Sciences, University of Warwick, Coventry CV4 7AL, U.K.; ‡ Department of Chemistry, University of Warwick, Coventry CV4 7AL, U.K.

## Abstract

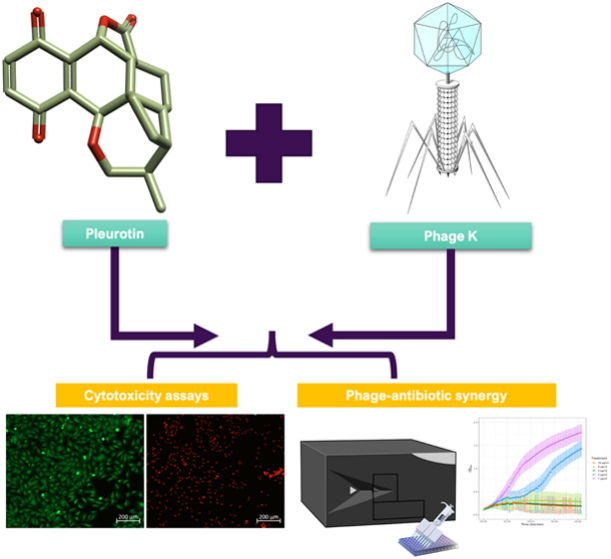

The surge in antibiotic-resistant *Staphylococcus aureus* infections has been deemed a major public health concern. There is an urgent need for novel antimicrobial therapies, chemical and nonantibiotic. The basidiomycota-derived, secondary metabolite pleurotin has been shown to be effective against Gram-positive bacteria, while bacteriophages could be the ultimate nonantibiotic alternative. In this study, the combination of pleurotin and phage K targeting *S. aureus* was examined. Pleurotin was isolated from the basidiomycota fungus *Hohenbuehelia grisea*. The cytotoxicity of pleurotin was assessed in two human cell lines in comparison to pleuromutilin, vancomycin, and phage K. The antibiotics were then tested independently or in combination with phage K against two *S. aureus* strains. Cytotoxicity of pleurotin in human cells was comparable to vancomycin and pleuromutilin. Results suggest that adding phage K has a synergistic effect and can lower the MIC for pleurotin, pleuromutilin, and vancomycin. This demonstrates that pleurotin could be a viable antistaphylococcal drug.

## 1 Introduction

The response to the rise of antimicrobial resistance has been lacking so far with the discovery of new antibiotic classes almost grinding to a halt over the last few decades.^1^ The ESKAPE pathogen *Staphylococcus aureus* is one such bacterium of particular concern due to its resistance potential and high healthcare burden. First detected in 1961, methicillin-resistant *S. aureus* (MRSA) is characterized by its various resistance mechanisms and virulence factors.^2,3^ While vancomycin is still generally effective in treating MRSA, resistance, dosing challenges and toxicity have led to a reconsideration of its usage. Other anti-MRSA antibiotics have become available, but clinical evidence supporting alternative treatments has not been robust or consistent enough to fully replace vancomycin as the standard therapy for these infections.^4,5^

**Figure 1:**
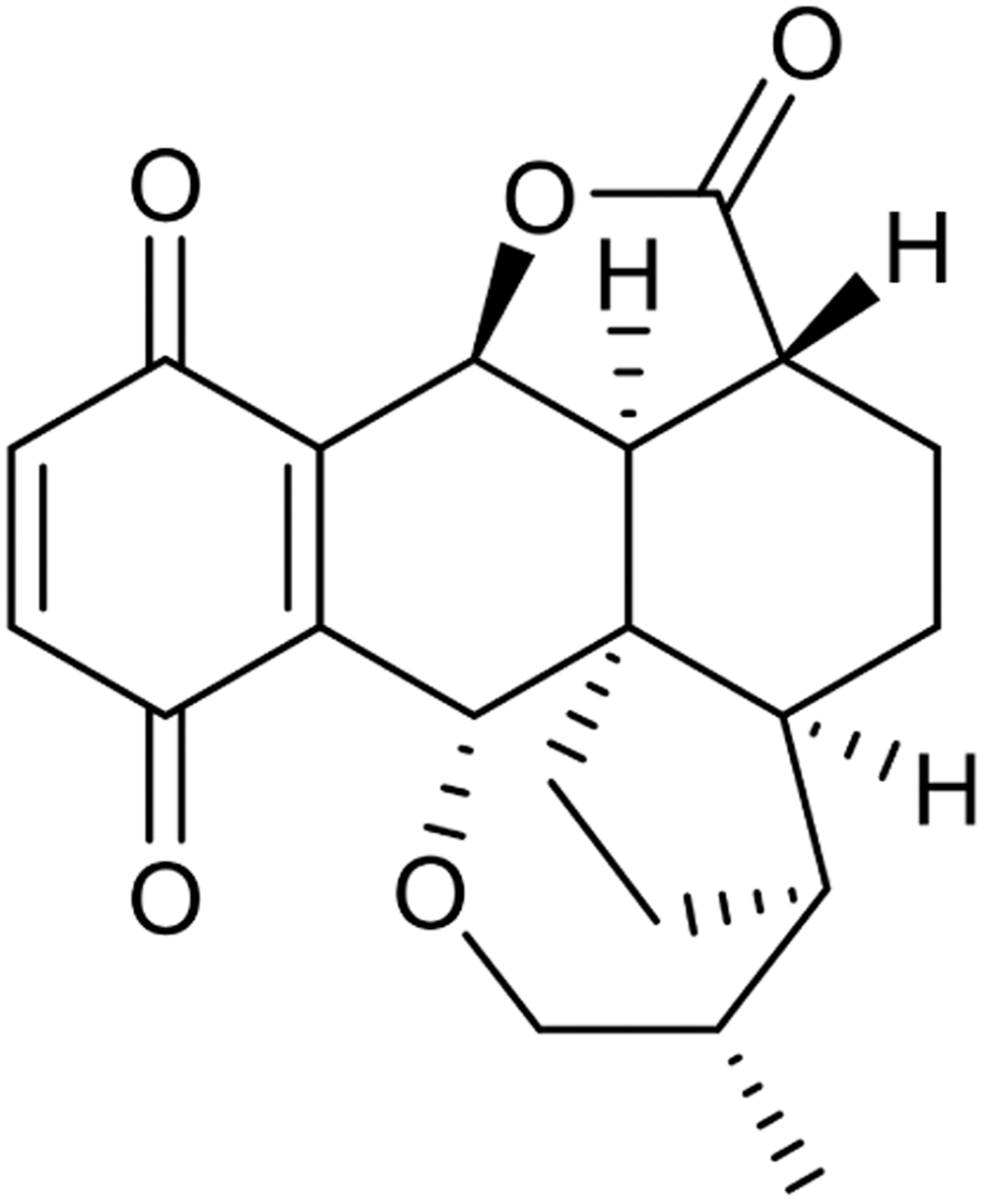
Pleurotin.

As such, the discovery of new classes with novel targets and mechanisms of action are urgently needed. This could be achieved through novel exploration, screening, and cultivation methods to find new microbial natural products from under-sampled ecological sources.^6^ With fungi being rich sources of interesting secondary metabolites, the meroterpenoid pleurotin (Figure 1), which was first isolated from the basidiomycota fungus *Pleurotus griseus* in 1947, has attracted attention recently for its anticancer properties.^7,8^ It works as an inhibitor on the thioredoxin-thioredoxin reductase (Trx-TrxR) system which plays a critical role in cellular DNA synthesis and oxidative stress, particularly affecting growth and colonization in dermatophytes.^9^ However, the system has also been identified to be a viable target in bacterial pathogens suggesting its antimicrobial potential with in vitro studies having also shown activity against Gram-positive bacteria.^10−14^

Yet, it is likely that even new classes of chemical antibiotics will only provide a temporary reprieve from the never-ending arms race to stay ahead of resistant bacteria. A broader therapy strategy that includes alternatives to conventional antibiotics is essential. Bacteriophages are the most abundant organisms on the planet, with an estimated 1 × 10^31^ phage particles in the environment and generally are easy to isolate providing a huge diverse reservoir of potential antimicrobial agents.^15^ However, there is also a risk of phage resistance emergence.^16,17^ This risk can be limited through combining phages with conventional antibiotics to achieve phage-antibiotic synergy (PAS), where sublethal concentrations of certain antibiotics resulted in increased phage production and lysis.^18−21^ Literature on phage-antibiotic combination (PAC) experiments that targeted *S. aureus* showed consistent synergistic results in vitro and in vivo depending on the type of antibiotic and phage used. Aside from having a bacteriocidal effect by itself, when matched well, phages can exert an adjuvant influence on previously diminished (older) antibiotics.^22−25^ However, it seems important to match the right antibiotic with phages, as studies have also reported other outcomes. Ideally, a synergistic effect is observed whereby combinatorial efficacy greatly exceeds that of any of the agents. However, results can be of an additive nature, where the sum of the individual effects of each agent is equal to the combinatorial efficacy. Indifferent, when the effect is less than the sum of each agent but more than an individual agent. Interference between the agents yields antagonism, reducing overall efficacy.^26^ However, anticipating these specific effects is challenging when considering the mechanisms of action of antibiotics.^25^

In this study, the antimicrobial therapeutic potential of pleurotin and its suitability for PAS were investigated against two *S. aureus* strains. First, considering its anticancer activity, the cytotoxicity of pleurotin was determined in two human cell lines and compared to two other antibiotics: vancomycin, which is clinically used against MRSA infections, and pleuromutilin, which is the progenitor of a novel class of clinically used antibiotics.^27^ Pleuromutilin is a basidiomycota-derived antibiotic, like pleurotin, and has shown activity against several Gram-positive bacteria, including MRSA and VRSA (vancomycin-resistant *S. aureus*). Semisynthetic derivatives of it have been developed to treat acute bacterial skin infections.^28,29^ It inhibits peptide bond formation and protein synthesis by binding to the peptidyl transferase component of the 50S subunit of ribosomes and no cross-resistance with other antibiotics has been observed.^28^ Subsequently, we looked into possible PAS through using the three antibiotics independently and in combination with phage K (a well-described staphylococcal lytic phage) in parallel experiments.^30^

## 2 Results

### 2.1 Purification of Pleurotin

Using the Applikon Biotechnology bioreactor, a stable culture of the pleurotin-producing*Hohenbuehelia grisea* was maintained. Pleurotin was detected in the supernatant crude extract in high amounts after 3 weeks and until week 10. Crude extracts were first fractionated using flash chromatography. Fractions containing high intensity peaks of pleurotin were then further purified through preparative high performance liquid chromatography (HPLC). Enriched fractions were characterized and quantified using ^1^H (q)NMR analysis. Figures S1 and S2 show the LC-MS and NMR spectra of the HPLC purified pleurotin fraction derived from the 3-week crude extract. This batch resulted in a yield of 6.4 mg/L. Shipley et al. achieved a titer of 1–2 mg/L using potato dextrose broth in their shake flask fermentations. They achieved significantly higher titers when using wood fiber (Silvacell), which was unfortunately unavailable to us.^31^

### 2.2 Pleurotin’s Cytotoxicity to Human Skin Fibroblasts is Comparable to That of Vancomycin and Pleuromutilin

To investigate the safety of pleurotin for cutaneous use, the overall cytotoxicity of pleurotin, phage K, vancomycin and pleuromutilin was tested against human skin fibroblasts and compared to T24 epithelial bladder carcinoma cells. A lactate dehydrogenase (LDH) assay and cell viability fluorescence stain were conducted after a 24 h incubation with the different treatments. LDH, an enzyme found in the cytosol of various cell types, is released into the cell culture medium when the plasma membrane is compromised. For the evaluation of the antibiotics, the MICs and/or slightly higher concentrations were used (pleurotin = 32–8 μg/mL, vancomycin = 4 μg/mL, pleuromutilin = 0.13 μg/mL). Cells were visualized using fluorescence microscopy after the cell viability fluorescence assay.

**Figure 2:**
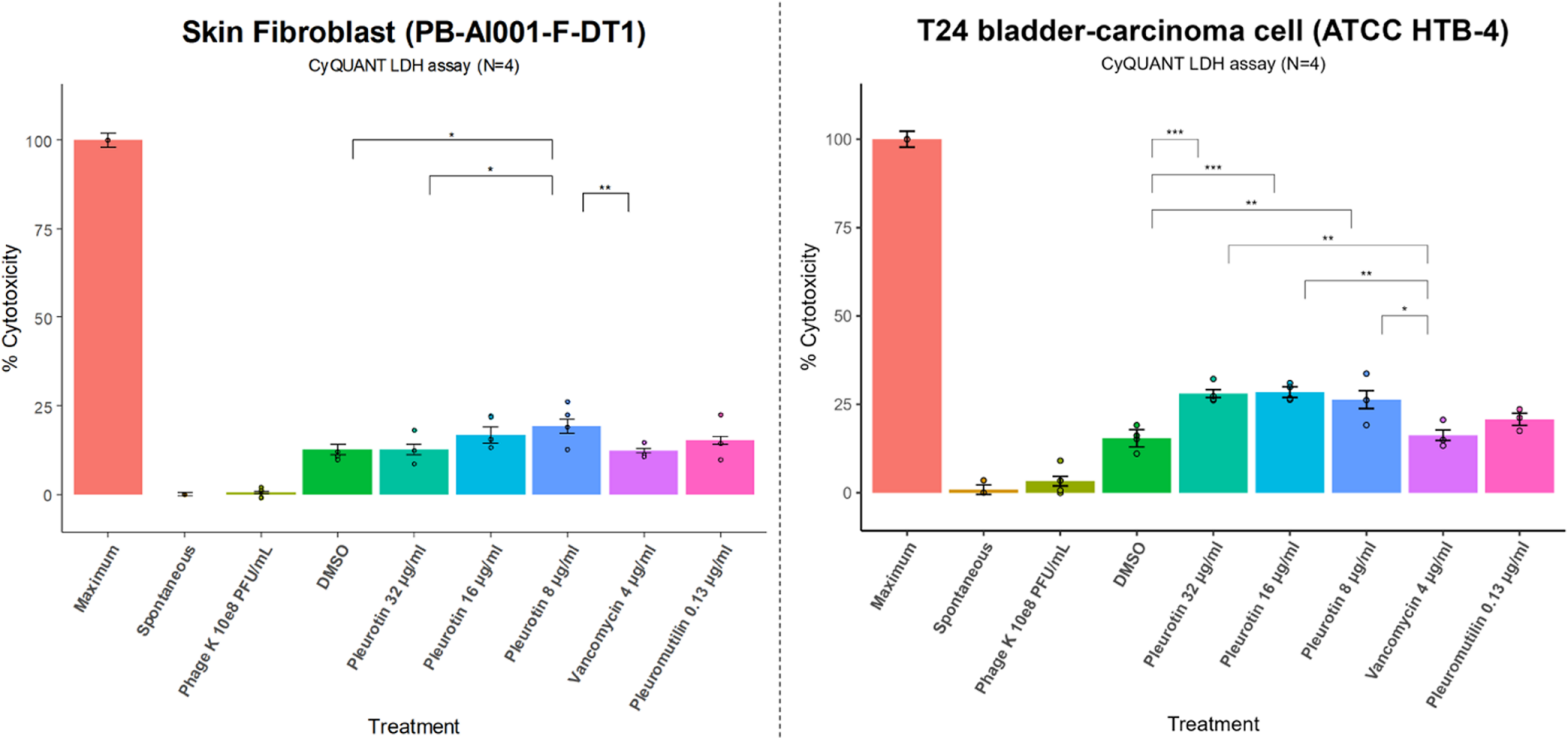
Cytotoxicity evaluation of pleurotin to human skin fibroblasts (PB-AI001-F-DT1) and T24 bladder-carcinoma cells (ATCC HTB-4). CyQuant LDH Cytotoxicity Assay. Maximum = maximum lysis buffer (positive control). Spontaneous = 10 μL water (negative control). Optical density measured at 490 and 680 nm and % cytoxicity calculated. *N* = 4, error bars indicate mean ± standard error. Estimated Marginal Means with a Holm–Bonferroni adjustment was used for pairwise comparisons: * = *P* < 0.05, ** = *P* < 0.01, *** = *P* < 0.001.

**Figure 3:**
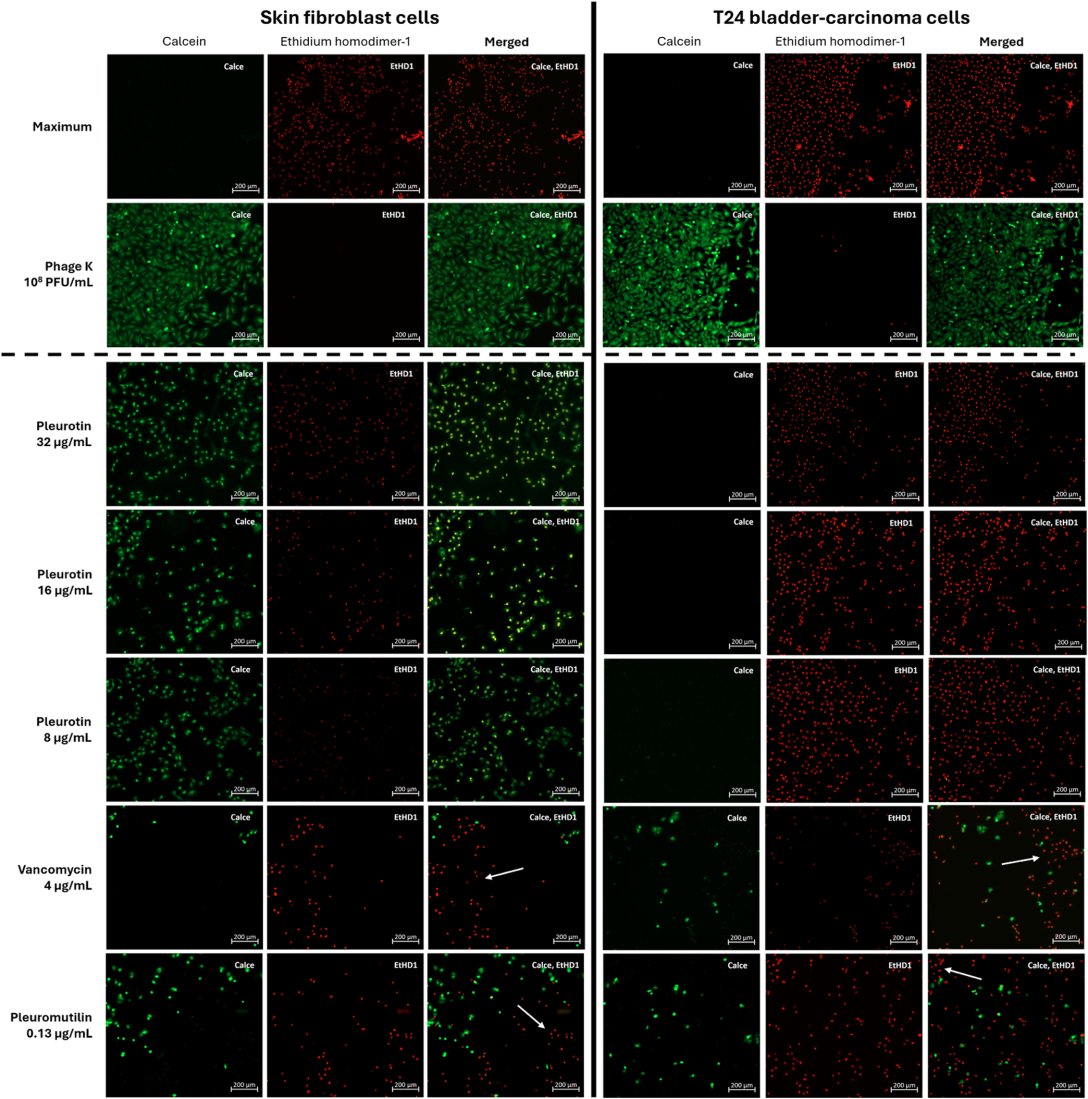
Live/dead cell viability fluorescence stain. Human skin fibroblasts (PB-AI001-F-DT1) and T24 bladder-carcinoma cells (ATCC HTB-4) stained with ethidium homodimer-1 (ex/em ∼495/∼515 nm) and calcein (ex/em ∼495/∼ 635 nm) from Invitrogen LIVE/DEAD Viability/Cytotoxicity Kit for Mammalian Cells. Ethidium homodimer-1 binds to nucleic acids in cells with damaged membranes and produces red fluorescence in dead cells. Green fluorescent calcein is converted from calcein-AM indicating esterase activity in live cells. Maximum = maximum lysis buffer (positive control). Spontaneous = 10 μL water (negative control). White arrows show localized cluster of cell death. *N* = 4.

The negative control was water, to account for any spontaneous LDH activity, and the positive control was a maximum lysis buffer from the LDH assay kit. A high dose of phage K at 1 × 10^8^ PFU/mL was used based on research evaluating human fibroblast and bladder cell viability when treated with staphylococcal phages.^32,33^ Phage K showed almost no cytotoxicity (0.5–3.2%) in the LDH assay in both cell lines (Figure 2) and that is also verified in the viability fluorescence imaging (Figure 3). The DMSO control exhibited some toxicity in the skin fibroblasts (12.6%) and T24 cells (14.2%). This is reflected in the fluorescence images by changes in morphology after 24 h where the cells were reduced in number, losing their star-like shape with fewer lateral protrusions and exhibiting increased plasma membrane damage (Figure 3 and S3). In the human skin fibroblasts, the LDH assay showed that cytotoxicity of the different antibiotic concentrations and DMSO ranged between 12.3 and 19.2%. While significant differences in cytotoxicity were detected between the different pleurotin concentrations and the DMSO control, they were relatively small (6.6% difference) (Figure 2). A difference was also observed between vancomycin and pleurotin at 8 μg/mL. There were no major differences in cell shape compared to the negative control (Figure S3). However, some level of membrane damage was present in pleurotin-treated cells and similarly to the DMSO control, cell density decreased, calcein (green) fluorescence intensity was reduced and EtHD1 (red) fluorescence was increasingly present. Interestingly, cell morphology seems to be at the start of a transition phase to a more compact appearance. This change was more apparent in the vancomycin (12.3%) and pleuromutilin (15.3%) treated cells. Live cells had compact shapes with barely any protrusions and green fluorescence intensity was comparatively high, whereas red fluorescence was more intense. Interestingly, live and dead cells were highly clustered. Overall, the data seems to suggest that the cytotoxicity of pleurotin in cutaneous cells is relatively low and comparable to pleuromutilin and close to vancomycin.

In contrast, slightly higher toxicity was present in the T24 carcinoma bladder cells. Pleurotin cytotoxicity levels increased to 26–29%. There was a slight increase, as well, for pleuromutilin (20.8%) and vancomycin (16.3%). However, fluorescence imaging reveals a more dramatic picture, especially for pleurotin. The 10% increase in pleurotin cytotoxicity compared to the skin fibroblasts constituted a more drastic change in morphology. Cell membrane damage was more pronounced with the majority of cells exhibiting increased EtHD1 fluorescence intensity. Similarly to cells in the positive control that are treated with the lysis buffer, cell shape was predominantly circular with hardly any protrusions indicating lack of surface adhesion and loss of cell viability. The pleuromutilin and vancomycin images show similar levels of cell viability with somewhat localized clusters of live and dead cells. The increased cytotoxicity of pleurotin can be explained by its ability to inhibit the thioredoxin-thioredoxin reductase (Trx-TrxR) system which play a key role in cellular oxidative stress response and redox signaling.^9^ It is overexpressed in several cancers and is involved in upregulating Hypoxia-induced Factor 1 (HIF-1), which promotes apoptosis resistance, angiogenesis, and other tumorigenic processes.^8,34^ Additionally, DMSO has been reported to suppress cancer proliferation, induce apoptosis and work as an adjuvant for a number of cancer therapies.^35^ These results suggest that pleurotin has a safety profile similar to vancomycin and pleuromutilin when used in a dermatological context whereas it has significantly higher cytotoxicity in carcinoma bladder cells, as expected.

### 2.3 Antibiotic Susceptibility Evaluation of Pleurotin against *S. aureus*

**Figure 4:**
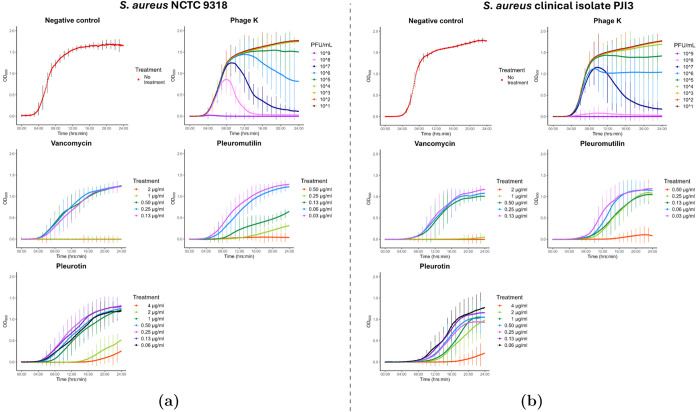
Antibiotic MIC determination: sublethal concentrations. 24 h growth curves (OD_600_) of *S. aureus* strains NCTC 9318 (a) and PJI3 (b). Sublethal concentrations of pleurotin (4–0.06 μg/mL, reported MIC < 33.3 μg/mL) were compared to vancomycin (2–0.13 μg/mL μg/mL, reported MIC = < 2 μg/mL) and pleuromutilin (0.5–0.031 μg/mL, reported MIC50/90 = 0.12/0.25 μg/mL), and the virility of phage K (10^9^–10^2^ PFU/mL). *N* = 3, error bars indicate mean ± standard deviation.

To ascertain sublethal concentrations, purified pleurotin was tested in a 2-fold serial dilution range against the *S. aureus*lab strain NCTC 9318 and the resistant clinical isolate PJI3 using the broth microdilution method as per the CLSI guidelines. Their genomes were screened for resistance genes using AMRFinderPlus. Surprisingly, it detected several AMR genes in both strains for chloramphenicol, erythromycin, fosfomycin and tetracycline and multidrug efflux pumps (Table S5). However, while NCTC 9318 is a reference and phage K propagating strain that should be methicillin-susceptible (MSSA), studies have shown that MSSA strains can exhibit resistance to antibiotics like erythromycin, tetracycline, and aminoglycosides.^36,37^ Using cefoxitin as a marker for methicillin susceptibility, MIC testing shows that *S. aureus* NCTC 9318 is sensitive to cefoxitin (MIC = 1 μg/mL), according to the EUCAST breakpoint guidelines (<4 μg/mL, Table S4).^38^ Clinical isolate antibiotic susceptibility testing at the University Hospital Coventry, recorded that PJI3 was resistant to clindamycin and intermediate for ciprofloxacin (Table S4).^33^ The antistaphylococcal activity of pleurotin was compared to phage K, vancomycin, and pleuromutilin. The OD was monitored at 600 nm for 24 h (Figures 4 and S4) and the MIC was determined to be the concentration that showed no growth after 24 h (Table 1). This allowed for capturing the effects of the antibiotics and phage on population density and any possible resistance population dynamics of the bacteria during treatment.

**Table 1:** Summary of MIC and Synergy Results against *S. aureus* Strains^a^

	MIC (μg/mL)					
	9318		PJI3		FICI	
antibiotics (reported MIC, μg/mL)	no phage	phage	no phage	phage	9318	PJI3
vancomycin ≥ 2	2	0.125	2	0.125	0.073	0.073
pleuromutilin 0.12–0.25	0.5	0.0313	0.5	0.0313	0.073	0.073
pleurotin ≥ 4	8	0.0625	16	0.0625	0.018	0.014

a FIC index: ≤ 0.5 = synergism, ≥ 4.0 = antagonism, and 0.5–4.0 = no interaction.

In a previous study, Sandargo et al. observed a MIC for pleurotin of 33.3 μg/mL against *S. aureus* DSM346.^14^*S. aureus* NCTC 9318 was susceptible to vancomycin (1 μg/mL), pleuromutilin (0.5 μg/mL) and pleurotin, with the MIC for pleurotin being 4-fold lower (>8 μg/mL) (Figure S4) than reported by Sandargo et al.^14^ Results were similar for PJI3 as it was susceptible to all three antibiotics. The main differences were a slightly higher MIC for pleuromutilin (>0.5 μg/mL) and pleurotin (16 μg/mL) (Figure S4). Phage K was tested in 10-fold serial dilution and showed expected effectiveness in reducing bacterial loads with a phage titer of 10^9^ PFU/mL resulting in total inhibition in both strains. With a multiplicity of infection (MOI) of 1 × 10^4^ (phage particles to a bacterial cell) resulting in almost total suppression of growth, the lack of any growth and the absence of resistant subpopulations emerging after 24 h suggest that the MOI could be lowered.

### 2.4 All Phage K-Antibiotic Combinations Exhibit Synergistic Interactions

**Figure 5:**
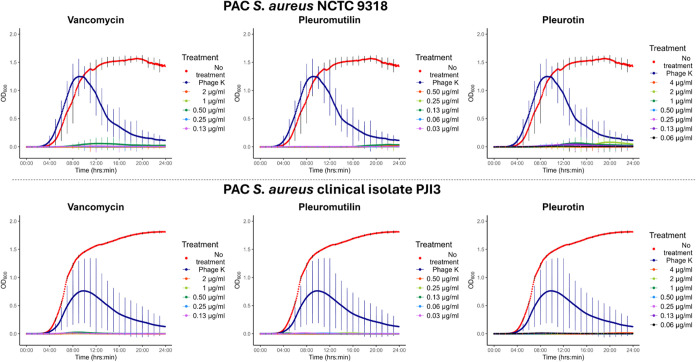
Phage-antibiotic combinations (PAC) against *S. aureus* NCTC 9318 and PJI3. 24 h growth curves (OD_600_) evaluating the combination of pleurotin (4–0.06 μg/mL, MIC = 8 μg/mL), vancomycin (16–1 μg/mL, MIC = < 2 μg/mL) and pleuromutilin (0.5–0.031 μg/mL, MIC50/90 = ≥ 0.5 μg/mL) with phage K (1 × 10^7^ PFU/mL). Phage K at 1 × 10^7^ PFU/mL alone is shown in dark-blue. *N* = 3, error bars indicate mean ± standard deviation.

Next, using pleurotin, vancomycin and pleuromutilin in parallel experiments, the synergistic effects of phage K were assessed and compared to phage and antibiotics alone. Pleurotin (4–0.06 μg/mL) and vancomycin (2–0.13 μg/mL) were further diluted to the sublethal concentrations established in the single agent assay. Pleuromutilin used the same range as the single agent experiment as most of the concentrations were already sublethal. 1 × 10^7^ PFU/mL of phage K (MOI = 10^2^) was added at the same time. The phage-antibiotic combinations were then incubated for 24 h and OD_600_ values were measured. The resulting growth curves are presented in Figure 5.

**Figure 6:**
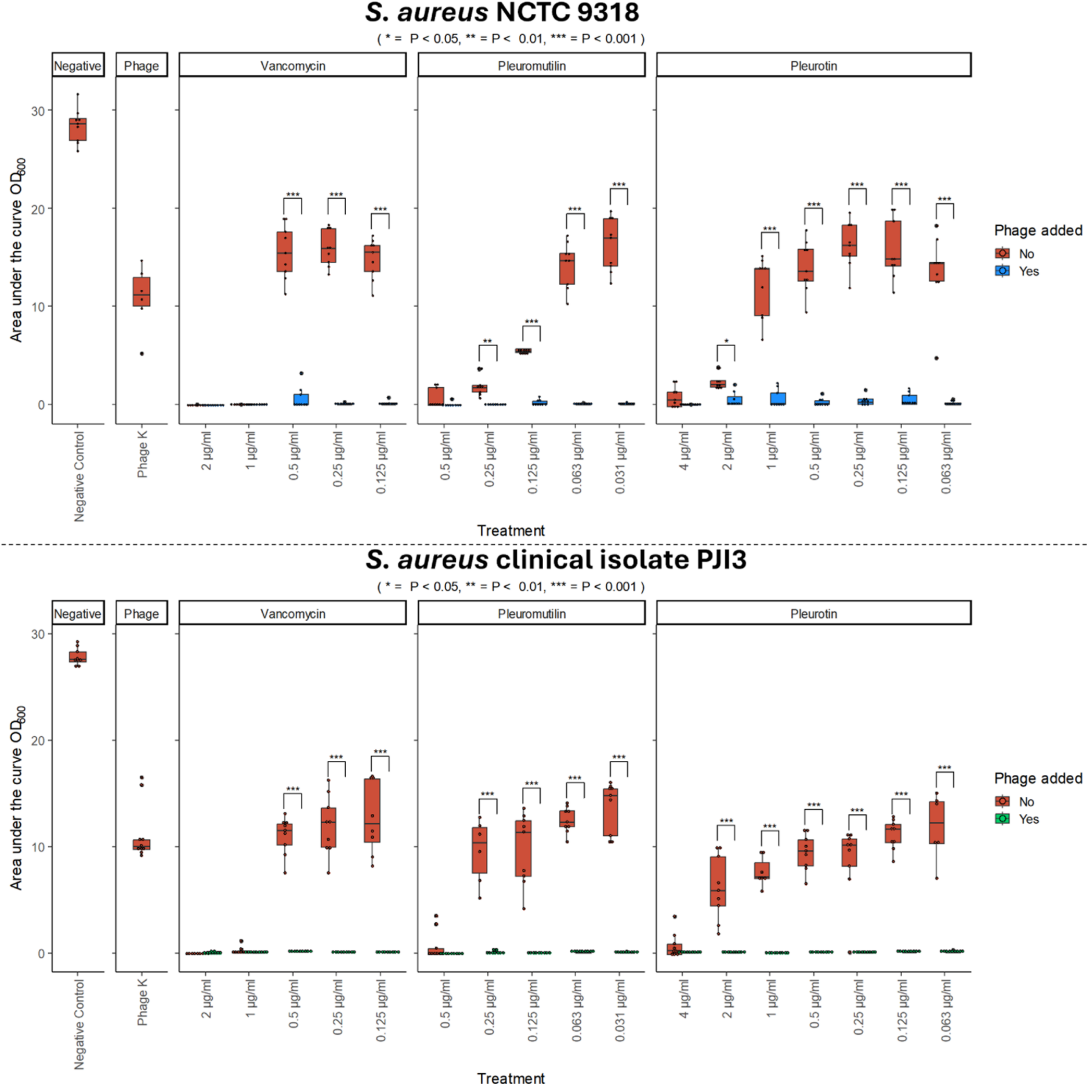
Quantification of bacterial reduction based on the area under the curve (AUC) of treated bacteria to compare the effects of using antibiotics alone vs phage-antibiotic combination. Phage K AUC at 1 × 10^7^ PFU/mL. *N* = 3. Estimated marginal means with a Holm-Bonferroni adjustment was used for pairwise comparisons: * = *P* < 0.05, ** = *P* < 0.01, *** = *P* < 0.001.

In addition, to enable comparisons between growth curves, the area-under-the-curve (AUC) for each treatment was calculated and plotted (Figure 6). Finally, interactions between phage and antibiotic were measured by calculating a fractional inhibitory concentration index (FICI) (Table 1). These were categorized into three conservative classifications based on the recommendations by Odds:^39^ (1) Synergism (FICI ≤ 0.5) occurs when the combined treatment results in a more significant bacterial reduction than the sum of either substance alone. (2) Antagonism (FICI ≥ 4.0) is evident when the combined treatment is less effective than the best-acting single agent in reducing bacterial levels. (3) No interaction (0.5 < FICI < 4.0) for outcomes that are neither synergistic or antagonistic. These can be additive when the combined treatment is equal to the sum of either substance alone or indifferent when the combined treatment is equivalent to the best-acting single agent.

The difference to the single agent experiment was pronounced. All phage-antibiotic combinations yielded synergistic interactions against both *S. aureus* strains with almost no growth being detected (Figure 5) with FIC indeces well below the 0.5 threshold (Table 1). The addition of phage K to vancomycin led to a 16-fold reduction in MIC (*p* < 0.001, Figure 6). For pleuromutilin this was the same (*p* < 0.001). The reduction was more pronounced in pleurotin which equated to a 128-fold reduction in NCTC 9318 (*p* < 0.001) and 256-fold reduction in PJI3 (*p* < 0.001).

## 3 Discussion

The main aim of this study was to investigate the antistaphylococcal potency of pleurotin and suitability for use, in combination with phage K, in cutaneous infections. Phage K (ATCC 19685-B1) is a well-known lytic phage from the *Myoviridae* family that targets a wide range of *Staphylococci*, with no reported bacterial toxins encoded in its genome.^30,40−43^ It has been used in several in vitro experiments and shown to be effective against *S. aureus* planktonic cells and biofilms, alone and in combination with antibiotics.^33,44−46^ We compared pleurotin to two clinically relevant antibiotics that are representative of their classes, vancomycin (glycopeptide cell wall synthesis inhibitor) and pleuromutilin (protein synthesis 50S subunit inhibitor).^28^ The antibacterial mechanism of action of pleurotin is currently unknown though it exhibits potent anticancer activity through inhibition of the Trx-TrxR system.^8^ The system appears crucial in the oxidative stress response of Gram-positive bacteria and therefore Trx-TrxR inhibitors have been proposed as a promising antibiotic class with bactericidal potential.^11^ So, there is a possibility that pleurotin might be part of this novel class if its antibacterial mode of action were found to be the same as its anticancer mode of action.

The cytotoxicity of phage K, vancomycin, pleuromutilin, and pleurotin was evaluated in human skin fibroblasts and bladder carcinoma cells. Phage K was well tolerated, hardly affecting cell viability. The results in skin fibroblasts show similar levels of cytotoxicity among the antibiotics (12.3–19.2%). According to the ISO 10993–5:2009 standard, a reduction in cell viability greater than 30% compared to untreated controls is considered cytotoxic.^47^ The only significant differences between antibiotics were observed between pleurotin and vancomycin, in both cell lines (*P* < 0.01), where the percentage point difference was 6.9% (19.2–12.3%) in skin cells and 10.1% (26.2–16.3%). For the bladder carcinoma cells, the cytotoxicity of pleurotin was slightly increased with a range of 26–29%, which can be explained by the anticancer properties of pleurotin. Few in vitro studies have explored the effects of various antibiotics on different cell types, so it is difficult to assess if the percentage point difference between pleurotin and vancomycin leads to biologically relevant effects on a tissue level. Vancomycin is known to be nephrotoxic and some in vitro studies have shown that vancomycin can be cytotoxic to human osteoblasts, skeletal muscle cells, dermal fibroblasts, and keratinocytes, though at longer exposure times and higher concentrations than what was used in this study.^48−53^ When it comes to staphylococcal infections, vancomycin is used to treat cutaneous infections, bone infections, respiratory tract infections, and septicemia. Pleuromutilin itself is not used clinically, but derivatives like retapamulin and lefamulin have been approved for human use.^29,54^ Similarly to vancomycin, in vitro research on cytotoxicity in human cell lines is limited, making comparisons difficult. Nevertheless, clinical trial studies show that lefamulin is well tolerated in patients.^29^ A study by Zhang et al. tested the cytotoxicity of a pleuromutilin derivative and showed that cell viability was around 90% in human kidney cells, which is comparable to our pleuromutilin LDH results (15.3 and 20.8% cytotoxicity in skin fibroblast and bladder cells, respectively). Our LDH assay results indicate that cytotoxicity is under the threshold set by the ISO 10993–5:2009 standard for all treatments, though morphological changes were observed in some conditions suggesting cells were experiencing stress. In vivo research will be necessary, as monolayer cell cultures are limited models that do not completely mirror the complex tissue environment and interactions with other bioactive molecules in the extracellular matrix (ECM).^55,56^

The PAC growth curves revealed that antistaphylococcal activity might be improved by using sublethal concentrations that could then stimulate phage-mediated killing, implying that concentrations can be diluted even further. This is especially beneficial for pleurotin as it allows for reducing the impact of its cytotoxic activity, which while comparable to vancomycin and pleuromutilin, could still lead to significant side-effects. The results obtained for vancomycin are in agreement with previous research on phage-vancomycin synergy.^23,24^ Intriguingly, the addition of phage to pleuromutilin, a protein synthesis inhibitor, yielded a synergistic result.^28^ However, an antagonistic effect was expected as demonstrated in previous research using antibiotics that inhibit bacterial protein synthesis.^25,26,57−60^ These showed that combinations of these classes of antibiotics with phages could lead to limited phage propagation. Our findings could be explained by the concentration of phage K used (MOI = 10^2^) possibly resulting in an abundance of phage particles, such that lysis happens without the need for propagation. This suggests that a lower phage MOI or higher concentrations of pleuromutilin could lead to either decreased effectiveness or even a switch to an antagonistic interaction.

In conclusion, to the best of our knowledge, no studies have been conducted until now involving the combination of phages with pleurotin or pleuromutilin against *S. aureus*. Our study showed that pleurotin is a promising candidate for PAS applications with an acceptable level of cytotoxicity given its anticancer properties. Further studies would need to look into their effectiveness in combination with phage against MRSA and vancomycin-resistant strains. Also, to understand the underlying mechanism of the PAS observed in pleurotin and pleuromutilin, the interplay between concentrations of phage and timing of administration need to be investigated in more depth. Furthermore, other antistaphylococcal phages could be tested, individually or in cocktails.

## 4 Methods

### 4.1 Pleurotin Production

*Hohenbuehelia grisea* ATCC 60515 was cultured in a bioreactor in Yeast/Mold broth. The presence of pleurotin in the crude extract was detected using LC-MS. Flash chromatography and reversed phase HPLC were used to fractionate crude extracts. Fractions were screened for the presence of pleurotin. Pleurotin was isolated based on UV absorption (220 nm), collected in glass vials prior to being pooled and concentrated in vacuo. Purified pleurotin was structurally characterized and quantified using ^1^H (q)NMR based on spectra from Weaver et al.^61^ Detailed fermentation, fractionation, MS and NMR spectroscopy methods are described in the Supporting Information.

### 4.2 Bacterial Strains

*Staphylococcus aureus* subsp. aureus Rosenbach was obtained from the American Type Culture Collection (NCTC 9318, ATCC 19685) and used as a model strain for propagation of phage K (NCTC 9318) and bioactivity assays.^62^ Methicillin susceptibility of NCTC 9318 was determined by testing the minimum inhibitory concentration of cefoxitin using the broth microdilution method. A resistant clinical isolate of *S. aureus*, PJI3, isolated from a patient with a postorthopedic surgery infection was provided for testing by University Hospital Coventry pathology services (previously used and characterized in Burton et al.) (Table S4).^33^ The NCTC 9318 genome was downloaded from genomes.atcc.org and the PJI3 genome from the NCBI database (ASM3719570v1). Both genomes were run through AMRFinderPlus (Version 3.11.14) to detect the presence of possible antimicrobial resistance (AMR) genes (Table S5).

### 4.3 Phage Propagation and Enumeration

Propagation of bacteriophage K (ATCC 19685-B1) was performed using its host strain, *S. aureus* NCTC 9318, in Lysogeny Broth (LB). 400 mL of media was inoculated with 10 mL of the host strain overnight culture and incubated at 37 °C and 200 rpm until it reached an OD600 of 0.15–0.2. Subsequently, 10 mL of existing bacteriophage lysate was added with 2 mM MgSO_4_/CaCl_2_. The culture was then incubated for another 5 h at 37 °C and 200 rpm for 5 h. The culture was transferred to 50 mL falcon tubes and bacterial debris was pelleted by centrifugation at 4000*g* for 10 min at 4 °C. Afterward, the supernatant was collected into a 500 mL Duran bottle and 1 M NaCl was added to incubate at 4 °C overnight. Finally, the supernatant was sterilized using a 0.22 μm bottle top vacuum filter with a capacity of 500 mL (Corning).

Detection and exact enumeration of phage K particles was performed in triplicate, using the small drop assay method with its *S. aureus* host strain using a total of eight serial 10-fold dilutions of the phage’s stock in LB broth and BHI Agar supplemented with 2 mM MgSO_4_/CaCl_2_ as underlay agar.^63^

### 4.4 Phage Purification Using Ultrafiltration

For human cell assays, 400 mL of phage K lysate was purified to remove potential bacterial endotoxins. First, 40 g of PEG8000 (final conc. 10% w/v) was added to the solution and incubated at 4 °C for 2 h. The PEG8000-phage solution was transferred to 250 mL centrifuge bottles and high-speed centrifuged at 4 °C at 25,000*g* for 60 min in the Avanti JXN-26 high-speed centrifuge (Beckman Coulter) to precipitate phage particles. The phage pellets were resuspended in 20 mL SM 2 buffer (per 1 L: 100 mM NaCl, 8 mM MgSO_4_ × 7H_2_O, 25 mM Tris-HCl) and sterilized with a 0.2 μm membrane filter. The suspension was ultrafiltrated using 100 kDa Amicon Ultra-15 centrifugal units (UFC910024, Merck) in a Centrifuge 5804 R benchtop centrifuge (Eppendorf) at 3000g for 40 min in 10 min intervals to prevent the upper reservoir from drying up. The phages were washed until the flow-through was clear. After, 10 mL SM 2 buffer was added and the filter unit tube was vortexed horizontally to detach phages from the filter membrane. The purified phage was transferred to a sterile 15 mL centrifuge tube and stored at 4 °C.

### 4.5 Cytotoxicity Evaluation in Human Cells

#### 4.5.1 Tissue Cell Culture

Human skin fibroblasts cells (PB-AI001-F-DT1, PELOBiotech) and T24 epithelial urinary bladder carcinoma cells (ATCC HTB-4) were cultured in uncoated T75 flasks containing Dulbecco′s Modified Eagle Medium (DMEM) (Gibco) supplemented with 10% v/v Fetal Bovine Serum (FBS) (Labtech International, U.K.) and 1% v/v Penicillin-Streptomycin and were maintained at 37 °C in 5% CO_2_ under a humidified atmosphere.

#### 4.5.2 Lactate Dehydrogenase (LDH) Assay

Cytotoxicity to human cells was measured using the CyQUANT LDH Cytotoxicity Assay Kit (Invitrogen), using the protocol supplied with the kit. The skin fibroblast cells were grown up until ∼80% of confluency was reached. Cells were then trypsinised and resuspended in fresh media in 96-well plates (CytoOne, Starlab) with a final concentration of 5000 cells/mL and grown overnight. The next day, 10 μL of purified phage K (1 × 10^8^ PFU/mL) and 5 μL of pleurotin were added to a final volume of 100 μL. For positive controls/comparison, 5 μL of vancomycin and pleuromutilin were also added. The antibiotics were dissolved in cell culture grade DMSO (Sigma-Aldrich). Dilutions around reported MIC breakpoints for susceptible strains were used for pleurotin (32, 16, 8 μg/mL, MIC < 33.3 μg/mL),^13^ vancomycin (4 μg/mL, MIC = <2 μg/mL)^38^ and pleuromutilin (0.13 μg/mL, MIC50/90 = 0.12/0.25 μg/mL).^64^ A negative control with only 10 μL sterile water, a control with 5 μL DMSO and the maximum lysis positive control were also included. Plates were incubated for 24 h at 37 °C with 5% CO_2_.

All samples were measured using OD at 490 and 680 nm in a TECAN Spark plate reader (Tecan, Spark control V3.2). LDH activity was calculated by subtracting the OD_680_ (background signal from instrument) value from the OD_490_ value. The % cytotoxicity was then calculated using the following formula
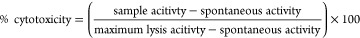
All experiments were done in technical triplicate and *N* = 4. A one-way ANOVA was conducted to determine the effects of the treatments on % cytoxicity using R with the tidyverse (v2.0.0), gcplyr (v1.10.0), and emmeans (v1.10.3) packages. All posthoc pairwise comparisons, between the different antibiotics and DMSO control were performed using emmeans in R with a Holm–Bonferroni adjustment applied.

### 4.6 Cell Viability Fluorescence Assay

After the supernatant was transferred in the LDH assay, the remaining, unused cells were used to conduct a viability/cytotoxicity stain using the LIVE/DEAD Viability/Cytotoxicity Kit for Mammalian Cells (L3224, Invitrogen). This would allow for a visual confirmation of the cytotoxicity results. The DMEM media was removed and the cells were washed two times with Dulbecco’s phosphate-buffered saline (DPBS) (Gibco) and 100 μL DPBS was added to the wells. The kit works by discriminating live from dead cells by simultaneously staining with red-fluorescent ethidium homodimer-1 to indicate loss of plasma membrane integrity and green-fluorescent calcein-AM which indicates intracellular esterase activity. The stock solution of both dyes were diluted in 10 mL of DPBS for a working solution of 2 μM calcein AM and 4 μM EthD-1. 100 μL of working solution was added directly to each well for a final volume of 200 μL. Samples were visualized using the Celldiscoverer 7 automated microscope (Zeiss) at 5× magnification using the ethidium homodimer-1 and calcein fluorescence filter profiles. Image acquisition was performed using Zeiss ZEN software. All experiments were done in technical triplicate and N = 4.

### 4.7 Pleurotin MIC Determination and Single Agent Comparison

The minimum inhibitory concentration of pleurotin against *S. aureus* NCTC 9318 and PJI3 was determined and compared to phage K, vancomycin and pleuromutilin using the broth microdilution method in lysogeny broth (LB). 2-fold serial dilutions of pleurotin (128–0.063 μg/mL), vancomycin (16–0.013 μg/mL) and pleuromutilin (0.5–0.031 μg/mL) were prepared from stock solutions and dissolved in DMSO. 10-fold serial dilutions of phage K lysate were prepared from approximately 1 × 10^9^ to 1 × 10^2^ PFU/mL per well. Overnight cultures of *S. aureus* NCTC 9318 and PJI3 were diluted to approximately 1.5 × 1 × 10^5^ CFU/mL and transferred to 96-well plates (CytoOne, Starlab). Twenty μL of each treatment was added to each well for a final volume of 200 μL. The plates were incubated at 37 °C with shaking, and the optical density (OD_600_) was measured every 5 min for 24 h in a TECAN Spark plate reader (Tecan, Spark control V3.2). All experiments were done in biological and technical triplicate.

### 4.8 Synergy Evaluation between Phage K and Antibiotics

The effect of combining phage K with pleurotin and the aforementioned antibiotics was tested with the broth microdilution method described above using sublethal concentrations. Phage K lysate was adjusted to approximately 1 × 10^7^ PFU/mL per well for an MOI (multiplicity of infection) of 100. For the PAS assay, 20 μL of phage and 20 μL of each antibiotic was added to each well for a final volume of 200 μL. The plates were incubated at 37 °C with shaking, and the OD_600_ was measured every 5 min for 24 h in the TECAN Spark plate reader. All experiments were done in biological and technical triplicate.

Linear mixed effects modeling was conducted to compare the effects of antibiotic concentration and phage treatment on the area under the curve (AUC) using R with the tidyverse (v2.0.0), gcplyr (v1.10.0), lme4 (v1.1–35.5) and emmeans (v1.10.3) packages.

The following formula was used: model = auc ∼ (1 |Replicate) + Treatment * phage-present. All posthoc pairwise comparisons, between the different antibiotic concentrations, with or without phage added, were performed using emmeans in R with a Holm–Bonferroni adjustment applied. Fractional inhibitory concentration indices (FICI) were calculated to analyze the interactions between phage and antibiotics using the following formula^39,65^
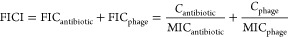
where *C*_antibiotic_ is the antibiotic concentration in combination in μg/mL and *C*_phage_ is the phage titer in combination in PFU/mL, MIC_antibiotic_ is the minimum inhibitory concentration alone and MIC_phage_ is the minimum inhibitory phage titer alone. Synergistic interactions are defined as FICI ≤ 0.5, antagonism as FICI ≥ 4.0 and an FICI between 0.5 and 4.0 is defined as additive/no significant interaction.

## Supporting Information Available

Additional experimental details and methods for chromatography; mass spectroscopy and NMR; supporting figures and tables: LC-MS and NMR spectra, *S. aureus* strain information; live/dead microscopy control images; MIC growth curves with lethal concentrations (PDF)
