# Supplementary material for: One-Two Punch: Phage-Antibiotic Synergy Observed against Staphylococcus aureus by Combining Pleurotin and Phage K
Source: ACS Omega. 2025 Mar 18;10(12):12026–36. doi: 10.1021/acsomega.4c09831 (PMC11966286; doi:10.1021/acsomega.4c09831)
Supplement: Supplementary file 1 — ao4c09831_si_001.pdf [file ao4c09831_si_001.pdf]

Supporting Information: One-two punch: Phage-antibiotic synergy observed against *Staphylococcus aureus* by combining pleurotin and phage K

Michaël Dagne Tadesse,† Nala Ali,† Martha White,† Lijiang Song,‡ Fabrizio Alberti,*,† and Antonia Sagona*,†

†*School of Life Sciences, University of Warwick, Coventry, CV4 7AL, United Kingdom*

‡*Department of Chemistry, University of Warwick, Coventry, CV4 7AL, United Kingdom*

E-mail: F.Alberti@warwick.ac.uk; A.Sagona@warwick.ac.uk

Contents

List of Figures **S-3**

List of Tables **S-3**

1 Material and methods **S-4**

1.1 Fungal fermentation and pleurotin extraction S-4

1.2 Pleurotin detection using LC-MS S-5

1.3 Crude fractionation using flash chromatography S-5

1.4 Fractionation using HPLC S-6

1.5 Structural characterisation using NMR spectroscopy S-7

2 Figures and Tables **S-8**

References **S-13**

List of Figures

S1 LC-MS detection of pleurotin: . . . S-8

S2 ^{1}H-NMR spectrum of pleurotin . . . S-9

S3 LIVE/DEAD Viability fluorescence imaging of spontaneous LDH activity (negative) and DMSO controls of skin fibroblasts and T24 cells: . . . S-11

S4 Antibiotic MIC determination: lethal concentrations . . . S-12

List of Tables

S1 LC-MS gradient protocol . . . S-5

S2 Biotage gradient protocol . . . S-6

S3 HPLC gradient protocol . . . S-6

S4 *S. aureus* PJI3 clinical isolate resistance profile & *S. aureus* NCTC 9318 methicillin susceptibility: . . . S-10

S5 AMRFinderPlus Detection of the presence of possible resistance genes in *S. aureus* strains: . . . S-10

1 Material and methods

1.1 Fungal fermentation and pleurotin extraction

Using *Hohenbuhelia grisea* ATCC 60515, several fermentations were done to biosynthesise pleurotin in sufficient amounts for downstream activity assays. The production method described in Shipley et al.[S1] was used as a starting point and adapted to our requirements. A pre-culture of *Hohenbuehelia grisea* (ATCC 60515) was grown in seed culture shake flasks at 24°C, 200 rpm, in the dark in 100 mL of YM liquid media consisting of Yeast Extract (3 g/L), Malt Extract (3 g/L), Peptone (5 g/L) and glucose (10 g/L) for 5 days. 100 mL of homogenised pre-culture was inoculated into 3.5 L of YM liquid media in an Applikon Biotechnology EZ-Control (Z310110010 Lab) Bioreactor System with a Applikon Bio stirred tank of 7 L (Getinge). The reactor was equipped with pH, DO and temperature sensors. The pH was set to 6.5 (+/- 0.5) and maintained using sulfuric acid (0.5 M H_2SO_4) and potassium hydroxide (1.72 M KOH). Oxygen levels were maintained using an air pump set at 3 mL/min. The temperature was set at 24°C and the stirrer speed at 180 rpm.

After 3 weeks, 1L of supernatant containing mycelia was extracted and a tissueruptor was used to homogenise the cultures. Then, 1 mL of 6M hydrochloric acid (HCl) was added per 1 L of culture to achieve a pH of ∼ 2-3. Ethyl acetate was added at a ratio of 1:1 to the cultures' volume, then poured into the flask and stirred with a magnetic flea for 15 minutes. The mix was poured into a separation funnel, shaken 6 times and left to rest for 15 minutes. Afterwards, the organic phase was collected. Anhydrous magnesium sulphate was added to the collected aqueous phase until the solution cleared and passed through filter paper (Whatman). The ethyl acetate was evaporated with the Rotavapor R-3 (VWR) using a 2L round bottom flask whilst suspended in a 25°C water bath. Once the ethyl acetate had evaporated, the residue left behind was re-suspended in 100% acetonitrile and placed in 2 ml amber glass vials to be stored at -20°C.

1.2 Pleurotin detection using LC-MS

To ascertain the presence of pleurotin and its congeners, reversed-phase LC-MS was performed on crude extract samples and fractions. The *Hohenbuehelia grisea* crude extracts were removed from storage and left to defrost in a dark room for one hour. Per sample, 70 μl was taken and added to a 630 μl mixture of 100% acetonitrile. This was then run through a 0.2 micron spin filter at 2000 x g for 1 minute. It was analysed using a UltiMate 3000 UHPLC System (ThermoFisher) coupled to an Amazon Speed ETD Iontrap MS (Bruker). A mixture of HPLC grade distilled water and HPLC gradient grade acetonitrile was used for a run of 60 minutes with a m/z range of 50-2200. Both solvents contained 0.1% formic acid. The column was kept at 25°C and a flow of 0.2 ml/minute was run through the Eclipse Plus C18 column (959758-902, 95Å, 2.1 x 100 mm, 1.8 µm, 1200 bar pressure limit, Agilent). Samples were run for 30 minutes in a gradient (Table S1). Data analysis was performed on Bruker Compass DataAnalysis software (version 4.2 Build 383.1)

Table S1: LC-MS gradient protocol

Time (min)	H2O (%)	Acetonitrile (%)
5	95	5
30	0	100
35	0	100
40	95	5
60	95	5

1.3 Crude fractionation using flash chromatography

Crude extract samples were fractionated using a high-performance automated flash chromatography system (Biotage® Selekt). In preparation, samples were run through a 0.2 micron spin filter at 2000 x g for 2 minutes. Using a Biotage® Sfär C18 6g reversed phase column, samples were run at a flow rate of 6 mL/min for 62 minutes in a gradient using HPLC grade distilled water and HPLC grade acetonitrile (Table S2). Fractions were collected based on absorbance at 220 nm with a threshold of 150 mAU, desiccated using the

EZ-2 Elite Solvent Evaporator (SP Genevac), resuspended in 100% acetonitrile and then subjected to LC-MS analysis. Fractions without any metabolites of interest were discarded.

Table S2: Biotage gradient protocol

Time (min)	H_2O (%)	Acetonitrile (%)
10	95	5
12	60	40
42	50	50
49	5	95
52	5	95
62	95	5

1.4 Fractionation using HPLC

FC fractions were further fractionated using a HPLC system (Agilent Infinity II 1260) with the Agilent OpenLab CDS software (Version 2.6). In preparation, fractions were run through a 0.2 micron spin filter at 2000 x g for 2 minutes. Using an Agilent C18 preparatory reversed phase column, fractions were run at a flow rate of 5 mL/min for 62 minutes in a gradient using HPLC grade distilled water and HPLC gradient grade acetonitrile (Table S3). Fractions were collected based on absorbance at 220 nm with a threshold of 150 mAU, desiccated, resuspended in 100% acetonitrile and then subjected to LC-MS analysis. Fractions without any metabolites of interest were discarded.

Table S3: HPLC gradient protocol

Time (min)	H_2O (%)	Acetonitrile (%)
10	95	5
12	60	40
42	50	50
45	0	100
55	0	100
62	95	5

1.5 Structural characterisation using NMR spectroscopy

Purified pleurotin was desiccated and subsequently dissolved in 700 μl deuterated chloroform ($CDCl_3$) and transferred into NMR tubes. NMR was performed on the Avance III 400 MHz instrument (Bruker).

For quantification, experiments were run using the methodology described in Muhamadejev et al.[S2]. This was done by building a standard curve with known concentrations of 1,3,5-trimethoxybenzene (TMB). This would be done to determine the $CHCl_3$ concentration in the $CDCl_3$ batch so the $CHCl_3$ peak can be used as an internal standard for sample quantitation. The 400 MHz Bruker Avance Neo was used with the following parameters: 14° pulse (90° pulse = 12 µs), d1 = 30 s, ns = 128, acquisition time = 4.19 s (32k points), spectral width 19.5333 ppm centered at 7.25 ppm. After quantification, samples were desiccated.

2 Figures and Tables

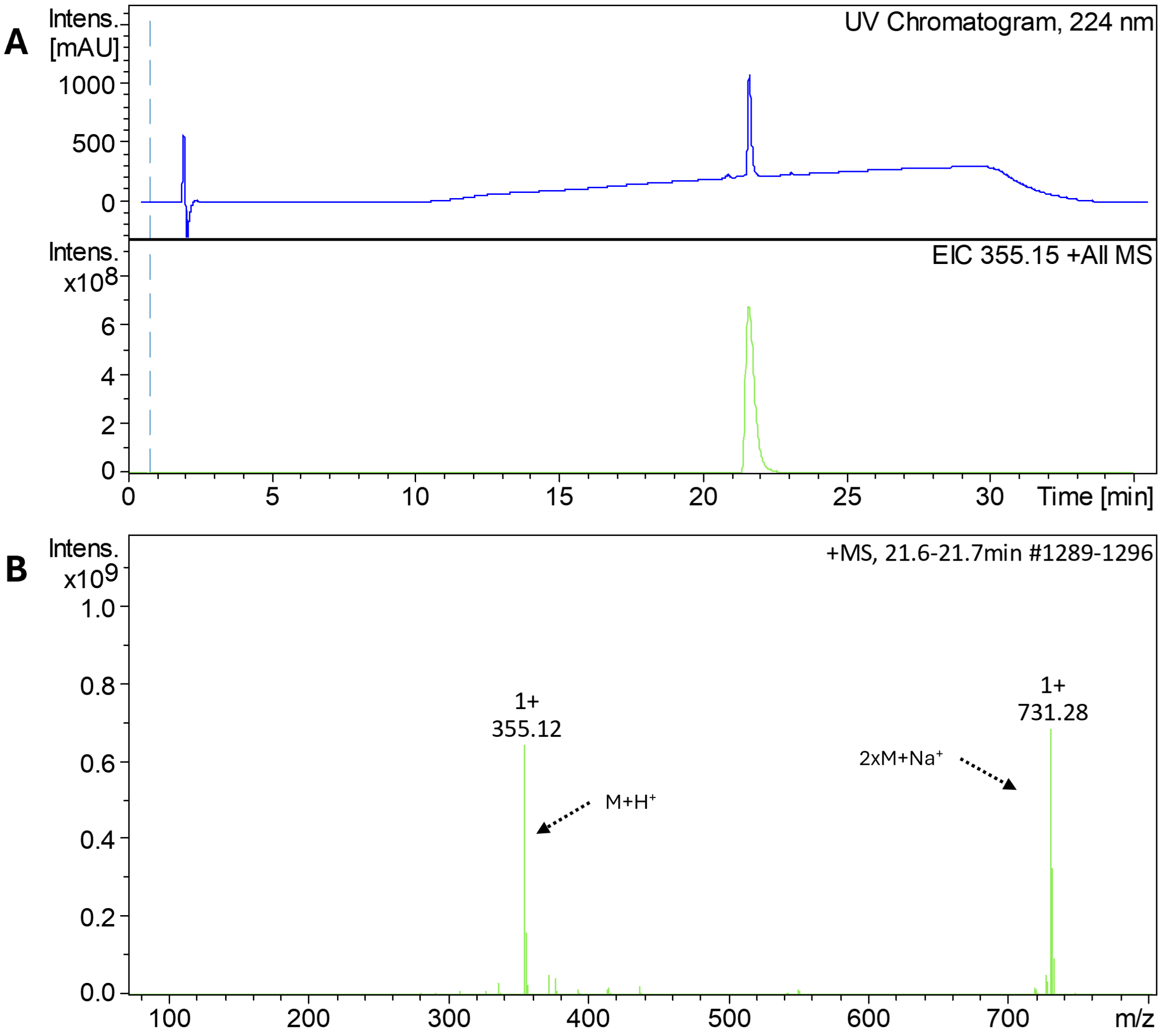

Figure S1: LC-MS detection of pleurotin: A) Extracted ion and UV chromatograms of HPLC fraction containing pleurotin is shown in positive mode at m/z = 355.1545 ±0.20 and UV 224 nm. Both show a singular major peak at retention time 21.7'. B) Mass spectrum of major peak with mass corresponding to pleurotin (M= $C_{21}H_{22}O_5$), expected m/z for $[M + H]^+ = 355.15$.

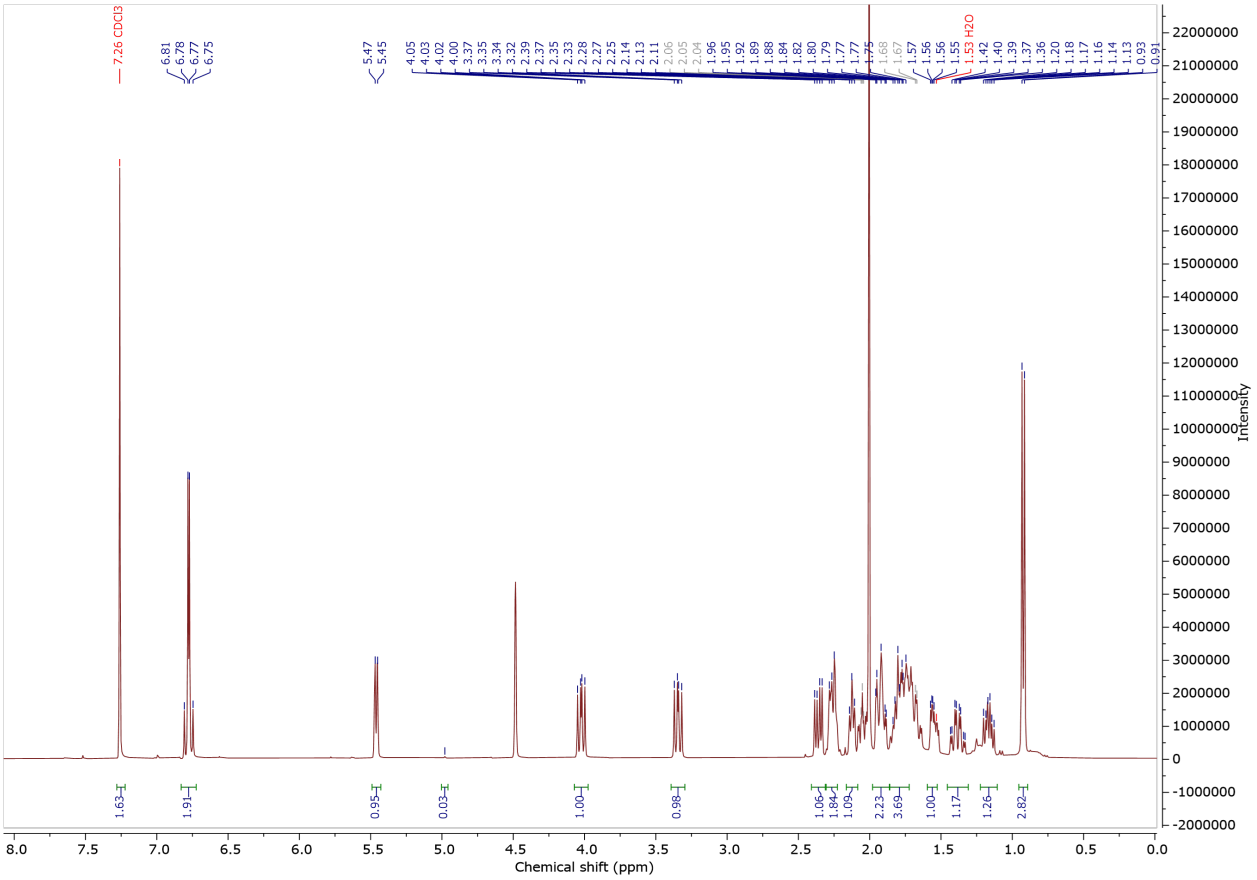

Figure S2: ^{1}H-NMR spectrum of pleurotin in $CDCl_3$ (400 MHz). Data in agreement with published ^{1}H-NMR spectra for pleurotin from Gao et al. (2024).[S3]

Table S4: *S. aureus* PJI3 clinical isolate resistance profile & *S. aureus* NCTC 9318 methicillin susceptibility:
Clinical isolate collected from University Hospital Coventry. NCTC 9318 methicillin susceptibility was assessed by determining the MIC of cefoxitin. (S) Susceptible, (I) Intermediate and (R) Resistant according to EUCAST breakpoint guidelines[S4]. Adapted from Burton et al. 2024[S5].

Strain	Antibiotic Susceptibility				
PJI3	Ciprofloxacin	Cotrimoxazole	Flucloxacillin	Fusidic Acid	Linezolid
	I	S	S	S	S
	Rifampicin	Teicoplanin	Vancomycin	Clindamycin	
	S	S	S	R	

NCTC 9318	Cefoxitin
	S

Table S5: AMRFinderPlus Detection of the presence of possible resistance genes in *S. aureus* strains:
AMRFinderPlus (Version 3.11.14) was used to screen the genomes

Strain	Gene symbol	Sequence name	Class
NCTC 9318	lmrS	multidrug efflux MFS transporter	Chloramphenicol/erythromycin
	fosB	FosB1/FosB3 family fosfomycin resistance bacillithiol transferase	Fosfomycin
	tet(38)	tetracycline efflux MFS transporter	Tetracycline
	mepA	multidrug efflux MATE transporter	Efflux
PJI3	erm(T)	23S rRNA (adenine(2058)-N(6))-methyltransferase	Macrolide
	lmrS	multidrug efflux MFS transporter	Chloramphenicol/erythromycin
	tet(38)	tetracycline efflux MFS transporter	Tetracycline
	mepA	multidrug efflux MATE transporter	Efflux

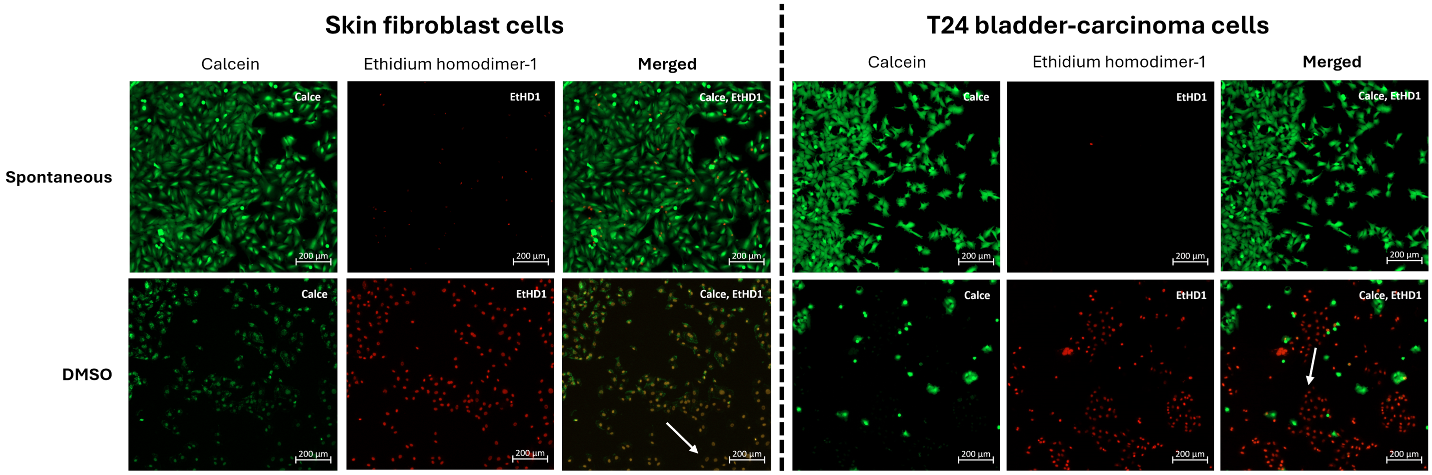

Figure S3: LIVE/DEAD Viability fluorescence imaging of spontaneous LDH activity and DMSO controls of skin fibroblasts and T24 cells: Calcein emission at 530 ± 12.5 nm, and EthD-1 at 645 ± 20 nm. Spontaneous = negative control (water), DMSO = dimethylsulfoxide without any antibiotics dissolved. White arrows show localised cluster of cell death. N = 4.

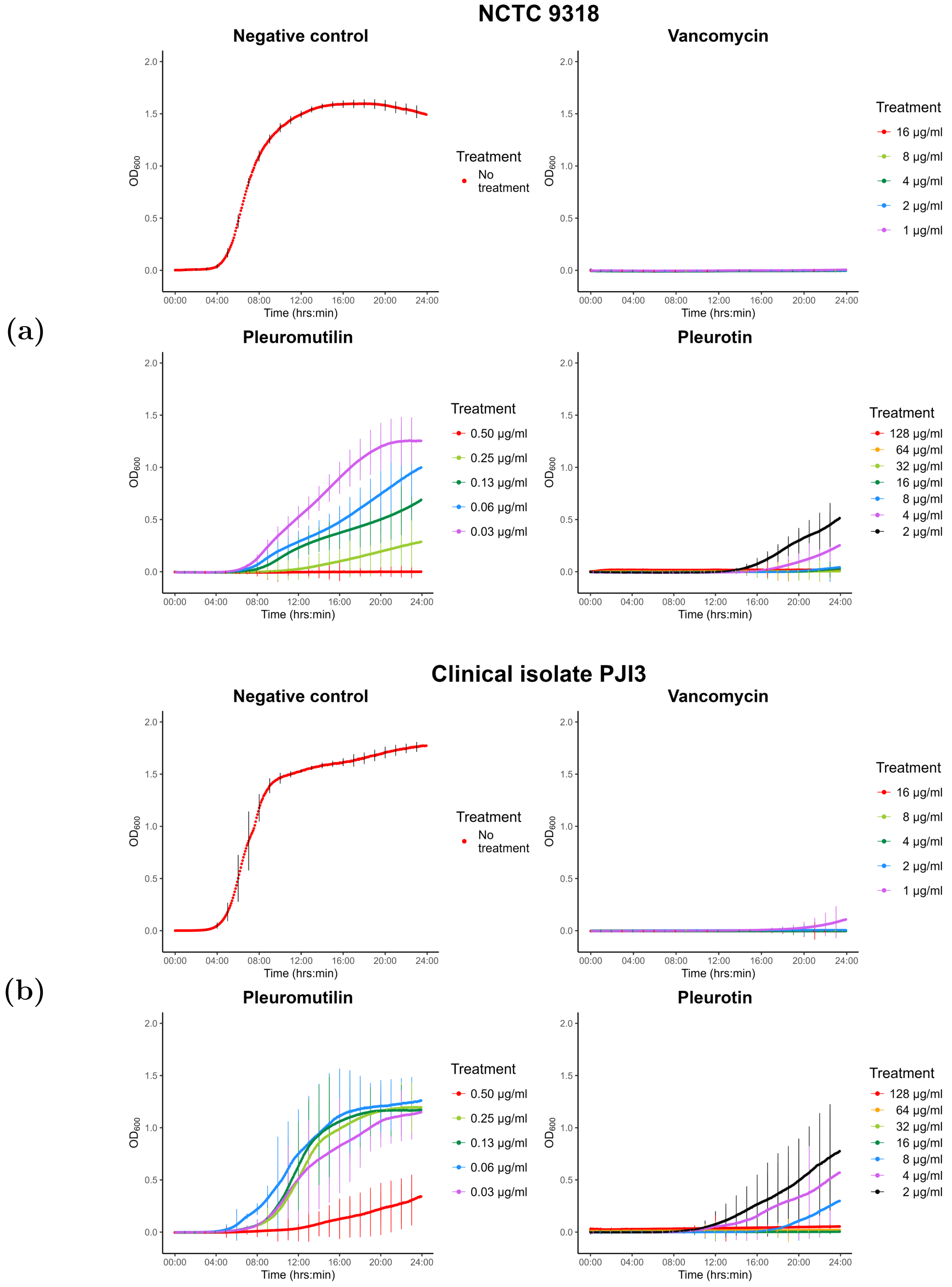

Figure S4: Antibiotic MIC determination: lethal concentrations. 24h Broth microdilution assays (OD600). *S. aureus* NCTC 9318 (a) and clinical isolate PJI3 (b) were subjected to lethal concentrations of pleurotin (128-2 µg/mL, reported MIC <33.3 µg/mL), vancomycin (16-1 µg/mL, reported MIC = <2 µg/mL) and pleuromutilin (0.5-0.031 µg/mL, reported MIC50/90 = 0.12/0.25 µg/mL). N = 3, error bars indicate mean ± standard deviation.

References

(S1) Shipley, S.; Barr, A.; Graf, S.; Collins, R.; McCloud, T.; Newman, D. Development of a process for the production of the anticancer lead compound pleurotin by fermentation of Hohenbuehelia atrocaerulea. *Journal of Industrial Microbiology and Biotechnology* **2006**, *33*, 463–468.

(S2) Muhamadejev, R.; Melngaile, R.; Paegle, P.; Zibarte, I.; Petrova, M.; Jaudzems, K.; Veliks, J. Residual solvent signal of CDCl3 as a qNMR internal standard for application in organic chemistry laboratory. *The Journal of Organic Chemistry* **2021**, *86*, 3890–3896.

(S3) Gao, Y.; Xia, Q.; Zhu, A.; Mao, W.; Mo, Y.; Ding, H.; Xuan, J. A Unified Synthetic Approach to the Pleurotin Natural Products. *Journal of the American Chemical Society* **2024**, *146*, 18230–18235.

(S4) EUCAST Clinical breakpoints-breakpoints and guidance (v 13.1). *European Committee on Antimicrobial Susceptibility Testing* **2023**, *1*.

(S5) Burton, N. J.; Melo, L. D. R.; Tadesse, M. F. D.; Pearce, B.; Vryonis, E.; Sagona, A. P. Isolation and characterisation of novel lytic bacteriophages for therapeutic applications in biofilm-associated prosthetic joint infections. *Sustainable Microbiology* **2024**, *1*, qvae028.
